# Therapeutic potential of small extracellular vesicles derived from lipoma tissue in adipose tissue regeneration—an in vitro and in vivo study

**DOI:** 10.1186/s13287-021-02291-z

**Published:** 2021-03-31

**Authors:** Pengyu Hong, Xiaoyang Xu, Xin Hu, Hao Yang, Yue Wu, Juan Chen, Kun Li, Zhangui Tang

**Affiliations:** 1 Department of Oral & Maxillofacial Surgery, Xiangya Stomatological Hospital & School of Stomatology, Central South University, Changsha, 410008 Hunan China; 2 Department of Oral and Maxillofacial Surgery, Xiangya Hospital, Central South University, Changsha, China

**Keywords:** Adipose tissue, Lipoma tissue, Small extracellular vesicles, Adipose tissue regeneration

## Abstract

**Objective:**

To explore the adipogenic effects of the small extracellular vesicles derived from the lipoma tissues (sEV-LT), and to find a new cell-free therapeutic approach for adipose tissue regeneration.

**Methods:**

Adipose tissue-derived stem cells (ADSCs) and small extracellular vesicles derived from the adipose tissues (sEV-AT) were isolated from human adipose tissue, while sEV-LT were isolated from human lipomatous tissue. ADSCs were characterized by using flow cytometric analysis and adipogenic and osteogenic differentiation assays. sEV was identified by electron microscopy, nanoparticle tracking, and western blotting. ADSCs were treated with sEV-LT and sEV-AT, respectively. Fluorescence confocal microscopy was used to investigate whether sEV-LT and sEV-AT could be taken by ADSCs. The proliferation and migration abilities and adipogenic differentiation assay of ADSCs were evaluated by CCK-8 assays, scratch test, and oil red O staining test, and the expression levels of adipogenic-related genes C/EBP-δ, PPARγ2, and Adiponectin in ADSCs were assessed by real-time quantitative PCR (RT-PCR). The sEV-LT and sEV-AT transplantation tubes were implanted subcutaneously in SD rats, and the neotissues were qualitatively and histologically evaluated at 2, 4, 8, and 12 weeks after transplantation. Hematoxylin and eosin (H&E) staining was subsequently used to observe and compare the adipogenesis and angiogenesis in neotissues, while immunohistochemistry was used to examine the expression and the distribution of C/EBP-α, PPARγ, Adiponectin, and CD31 at the 4th week.

**Results:**

The in vitro experiments showed that both sEV-LT and sEV-AT could be taken up by ADSCs via endocytosis. The scratch experiment and CCK-8 experiment showed that the migration area and proliferation number of ADSCs in sEV-LT group and sEV-AT group were significantly higher than those in the non-sEV group (*p* < 0.05). Compared with sEV-AT group, sEV-LT group had larger migration area and proliferation number of ADSCs (*p* < 0.05). Oil red O staining and RT-PCR experiments showed that, compared with the non-sEVs group, the lipid droplets and the mRNA expression levels of adipogenesis-related genes PPARγ2 and Adiponectin of ADSCs in sEV-LT group and sEV-AT group were significantly upregulated (*p* < 0.05); however, there was no statistical significance in the expression level of C/EBP-δ (*p* > 0.05). In addition, no significant difference in the amount of lipid droplets and adipogenesis-related genes between the sEV-LT groups and sEV-AT was seen (*p* > 0.05). At 2, 4, 8, and 12 weeks, the adipocyte area and the number of capillaries in neotissues in the sEV-LT groups and sEV-AT groups were significantly increased compared with the Matrigel group (*p* < 0.05); however, there was no dramatic difference between sEV-LT groups and sEV-AT groups (*p* > 0.05). At the 4th week, neotissues in the sEV-LT groups and sEV-AT groups all showed upregulated expression of C/EBP-α, PPARγ, Adiponectin, and CD31 protein, while neotissues in the Matrigel group only showed positive expression of CD31 protein.

**Conclusions:**

This study demonstrated that sEV-LT exerted promotion effects on adipose tissue regeneration by accelerating the proliferation, migration, and adipogenic differentiation of ADSCs in vitro and recruiting adipocytes and promoting angiogenesis in vivo. The sEV-LT could serve as an alternative cell-free therapeutic strategy for generating adipose tissue, thus providing a promising application prospect in tissue engineering.

## Introduction

Soft tissue defects resulting from resection of tumors, as well as from trauma and congenital abnormalities, not only lead to disfigurement, but also impair functions, making adipose tissue restoration an urgent clinical need [1]. In 1893, Neuber first reported the use of autologous fat transplantation to successfully repair tissue defects; however, controversial results were reported in the limited therapeutic effect of this strategy, due to fat reabsorption, necrotic, liquefaction, and possible scar contracture in the donor sites [2, 3]. In more recent years, studies have shown that transplantation of adipose tissue-derived stem cells (ADSCs) can enhance adipose tissue regeneration via the paracrine actions of various cytokines and growth factors [4–6]. For example, our previous studies have demonstrated that fat grafts consisting of platelet-rich plasma and ADSCs constitute an ideal transplant strategy, which may result in decreased absorption and accelerated fat regeneration [7]. Although cell-based therapy has demonstrated the beneficial effects on adipose tissue regeneration, there are still lots of problems in the application of those mesenchymal stem cells (MSCs), such as immunogenicity, low viability, and potential tumorigenic feature [8, 9]. Interestingly, recent works have demonstrated that paracrine factors significantly contribute to the therapeutic effect of stem cells on tissue repair [10]. In particular, extracellular vesicles (EVs) may play an important role in paracrine mechanisms and have attracted attention in basic research and clinical applications [11].

EVs are nano-sized membrane vesicles involved in intercellular communication, which have gained the most attraction as a potential and promising cell-free molecule used in clinical therapeutic applications [11–13]. According to the guideline of MISEV2018 (Minimal information for studies of extracellular vesicles 2018), extracellular vesicles with size less than 200 nm were termed as sEVs (small extracellular vesicles) [11]. The growing evidence has proved that sEVs are secreted by a variety of cells and mediate local and systemic intercellular communication by transfer their contents into the target cells [14]. In general, sEVs contain messages from the original cell sources including bioactive proteins such as cytokines and growth factors, as well as lipids and nucleic acids (RNA and DNA), which modulate biological behaviors of the target cells [15, 16]. Adipose tissue is an active endocrine organ that can secrete various factors to regulate adipogenesis via paracrine signals [17]. Previous studies have proved that sEVs derived from the adipose tissues (sEV-AT) are able to take part in a wide range of biological processes, especially for inducing adipogenic differentiation of ADSCs in vitro and promoting adipose tissue regeneration in vivo [17, 18].

Lipomas are common benign tumors of adipose tissues that originate in mesenchymal progenitors. Lipomas are usually treated when small in size; however, they sometimes can grow larger than 10 cm and can weigh over 1 kg [19]. Generally, most of the lipoma tissues after surgical resection are considered useless and used to be discarded. In clinic, intact lipoma tissues seem to be easier to obtain than normal adipose tissues, which are often fragmentized after liposuction. Studies have shown that a large number of adipocytes derived from lipoma tissues were strongly surrounded by Ki67+/CD34+ cells, indicating several altered biological activities such as proliferation, apoptosis, and stemness of those adipocytes [20, 21]. Although several researches have indicated that lipoma tissue may have a faster rate of adipogenesis than normal adipose tissue, there is no study about the effect of sEVs derived from the lipoma tissue (sEV-LT) on adipose tissue regeneration. Therefore, in this study, we examined and analyzed sEV-LT. We observed the effects of sEV-LT on the proliferation, migration, and adipogenic differentiation of ADSCs. Furthermore, we explored the inductive effect of sEV-LT on adipose tissue regeneration in subcutaneous chamber models of SD rats. Collectively, our study provides a novel theoretical basis and a potential cell-free therapeutic strategy for adipose tissue regeneration.

## Methods

### Animals and patients

Before surgery, all patients were informed of the purpose and procedures of this study and agreed to offer their excised tissues. Written consent was obtained from all participants involved in this study. Human adipose tissues and subcutaneous lipoma tissues were intactly obtained from male patients (mean age 36.7 ± 8.3 years, age range 25–50 years, *n* = 10) by surgical excisions, who underwent skin transplantations at Xiangya Stomatological Hospital (Changsha, China). SPF Sprague-Dawley (SD) rats were purchased from the Department of Laboratory Animals of Central South University (Changsha, China).

### Isolation and culture of ADSCs

ADSCs were obtained from human subcutaneous adipose tissues. The fresh adipose tissues were washed three times with sterile phosphate-buffered saline (PBS) containing 1% penicillin and streptomycin, chopped by sterile operation scissors into small pieces (1–2 mm^3^), and digested with 3 mg/ml type I collagenase (Sigma, Germany) for 40 min at 37 °C with shaking and then centrifuged at 1400 rpm for 7 min. The sediments were resuspended and expanded in the culture medium consisting of DMEM/F12 medium (BI, Israel), 10% fetal bovine serum (FBS, BI, Israel), 1% penicillin/streptomycin, and 2 mM L-glutamine at 37 °C with 5% CO_2_.

### Characterization of ADSCs

The ADSCs at passage 3 were harvested and counted. Approximately 1 × 10^5^ cells were washed and labeled with fluorescence-conjugated antibodies (Biolegend, USA) (CD105-PE, CD90-PE, CD73-PE, CD34-PE, CD14-PE) at room temperature for 30 min. Isotype control IgG1 and IgG2a were used to stain the cells as a control. After being washed with PBS twice, the fluorescence of ADSCs were observed. For adipogenic or osteogenic differentiation, the cells were seeded in standard 6-well tissue culture plates (1.5 × 10^5^ cells per well and incubated with adipogenic differentiation medium (Cyagen, China) for 1 week or with osteogenic differentiation medium (Cyagen, China) for 2 weeks, respectively. Then, the induced cells were stained separately with Oil Red O for 30 min to assess adipogenic differentiation or with Alizarin Red S (Cyagen, China) for 5 min at room temperature to visualize osteogenic differentiation.

### Isolation of small extracellular vesicles

Patients’ adipose tissues and lipoma tissues were minced into small pieces and transferred into a Celstir spinner flask (Wheaton) supplemented with Serum-free Dulbecco’s modified eagle medium/f12 (DMEM/F12) and 1% penicillin/streptomycin, respectively. The tissues were cultured at 37 °C and a rotational speed of 100 rpm for 2 days. The debris of tissues and cells were removed by centrifugation (2000*g*, 30 min). An additional centrifugation in Amicon® Ultra-50 Centrifugal Filter Units with Ultracel-3 membrane (3000Mw cutoff membrane, Millipore) at 5000*g* for 30 min was applied to concentrate lipoma tissue extract (LTE) and adipose tissue extract (ATE). Then, the ATE and LTE were mixed with the Total Exosome Isolation™ reagent (Life Technologies) at 4 °C overnight and a final ultracentrifugation step was performed at 10,000*g* for 1 h at 4 °C. The obtained pellet was resuspended in 400 μl of PBS and stored at − 80 °C with known concentration determined by using the Pierce BCA protein assay kit (KeyGEN, China).

### Characterization of small extracellular vesicles

The ultrastructure and size distribution of the sEV-LT and sEV-AT were analyzed by a transmission electron microscopy (TEM) (FEI Tecnai G2 Spirit, USA) and the ZetaView® system (Particle Metrix, Germany), respectively. Twenty-microgram vesicles was dissolved in RIPA Lysis Buffer (KeyGEN, China) and separated on polyacrylamide gels, blotted onto a nitrocellulose membrane. The specimens were then incubated with a primary antibody CD9 (1:1000, Abcam, ab92726), CD63 (1:1000, Abclonal, a5271), and TSG101 (1:1000, Proteintech, 28283-1-ap) at 4 °C overnight and followed by horseradish peroxidase-coupled secondary antibody for 1 h at room temperature. The labeled protein markers were visualized using ImageQuant LAS 4000 mini (GE Healthcare).

### Small extracellular vesicles labeling and cellular uptake

The sEVs were labeled with a membrane-labeling dye DiO (Invitrogen, USA) and then washed and resuspended in serum free DMEM/F12. After that, ADSCs were co-cultured with DiO-labeled vesicles for 6 h, washed with PBS three times, fixed in 4% paraformaldehyde, stained with phallotoxins (Invitrogen, USA), washed with PBS three times, counterstained with 4′,6-diamidino-2-phenylindole (DAPI, Sigma, Germany), and washed with PBS three times and imaged by a confocal microscopy (Olympus FV1000, Japan).

### Cell migration assay

The effects of sEV-LT and sEV-AT on ADSCs migration were evaluated in a scratch assay. ADSCs were seeded and cultured in 6-well plates at a seeding density of 2 × 10^5^ cells/well. When the cell confluence reached 90%, the medium was replaced with DMEM/F12 after washing with PBS twice, the confluent cell monolayer was scratched using a sterile 1000 μl pipette tip, and the cells were washed with PBS. The sEV-LT (40 μg/ml, 1.40 × 10^9^ particles/ml), sEV-AT (40 μg/ml, 2.55 × 10^9^ particles/ml), and an equal volume of PBS were added to the wells, respectively. Images were recorded at 0, 12, and 24 h after the monolayers were scratched. The migration area was measured by using ImageJ software and assessed as follows: migration area (%) = (A0 − An)/A0 × 100 (A0 represents the initial wound area (*t* = 0 h) and An represents the residual area of the wound at the time of measurement (*t* = n h)).

### Cell proliferation assay

The growth of ADSCs was determined by a Cell Counting Kit-8 (CCK-8, KeyGEN, China) assay. The ADSCs were cultured in 96-well plates with a seeding density of 1500 cells/well and the medium was replaced with PBS. The cells were cocultured with sEV-LT (40 μg/ml, 1.40 × 10^9^ particles/ml), sEV-AT (40 μg/ml, 2.55 × 10^9^ particles/ml), or an equal volume of PBS. At 0, 1, 2, 3, 4, and 5 days, 10 mL cell counting solution was added into each well and incubated at 37 °C for 1 h. The optical density (OD) was measured at 450 nm using a microplate reader (BioTek, USA).

### Adipogenic differentiation of ADSCs

The ADSCs at passage 3 were seeded at 2 × 10^5^ cells per well into six-well plates, cultured for 24 h, then rinsed with PBS and incubated with 2 ml of one of four different culture media for up to 14 days. The media used were as follows: (1) basal medium (DMEM/F12 supplemented with 10% FBS) as a negative control, (2) basal medium supplemented with sEV-LT (40 μg/ml, 1.76 × 10^9^ particles/ml), (3) basal medium supplemented with sEV-AT (40 μg/ml, 2.69 × 10^9^ particles/ml), and (4) adipogenic medium (DMEM/F12 supplemented with 10% FBS, 2 mM insulin+ 0.5 mM isobutylmethylxanthine + 0.1 μM dexamethasone + 5 μM rosiglitazone) as a positive control. The medium was changed every 3 days. The expression of adipogenic genes were analyzed by real-time quantitative PCR (RT-PCR) after 7 days of induction. After 14 days culturing, adipogenic differentiation was determined by the Oil Red O (Cyagen, China) staining. The Oil Red O in cells was extracted with 100% isopropanol for 15 min. The absorbance was measured at 520 nm with a microplate reader (BioTek, USA).

### Real-time PCR analysis

The TRIzol® reagent (Invitrogen, USA) was used to extract total RNA, which was reverse transcribed into cDNAs using the Revert Aid First Strand cDNA Synthesis Kit (Thermo Scientific, USA). The synthesized cDNAs were amplified with SYBR Premix ExTaq (TaKaRa Biotechnology, Japan) using a RT-PCR System (Biometra Tone, Germany). The PCR cycling parameters were 95 °C for 2 min, 44 cycles of 95 °C for 5 s, and 60 °C for 30 s (the primers used for RT-PCR are shown in Table 1).

**Table 1:** Primers used for RT-PCR

Gene	Primers
PPARγ2	5′-GCCCTTTGGTGACTTTATGGAG-3′
	GCAGCAGGTTGTCTTGGATGT
C/EBP-δ	CTGCCATGTATGACGACGAGAG
	CGCTTTGTGATTGCTGTTGAAG
ADIPONECTIN	CGTTCTCTTCACCTACGACCAGT
	ATTGTTGTCCCCTTCCCCATAC
GAPDH	TCAACGGCACAGTCAAGG
	ACCAGTGGATGCAGGGAT

### Adipose tissue regeneration experiments in an animal model

All operations were performed on 8-week-old SD rats (200 ± 20 g, *n* = 16) under general anesthesia (1% pentobarbital sodium, 10 mL/kg, intraperitoneal injection). A silicone tube with an internal diameter of 5.0 mm and a height of 5.0 mm was subcutaneously implanted into the longitudinal incision (about 4 cm) on the back of rats. The incision was closed with 4/0 nylon suture. Each rat was implanted with 3 tubes: (1) 100 μL Matrigel (Corning, USA) alone, (2) 100 μL Matrigel containing 100 μg sEV-LT by injection, and (3) 100 μL Matrigel containing 100 μg sEV-AT by injection. Rats were sacrificed at 2, 4, 8, and 12 weeks, respectively (*n* = 4 per time point), and their implanted tubes and contents were harvested and analyzed for further investigation.

### Histology

The excised neo-tissue samples from the silicone tubes were fixed in 10% formalin, dehydrated with a graded alcohol series, and embedded in paraffin. Sections with a thickness of 4 μm were stained with hematoxylin and eosin (H&E). Images of the histologic sections were examined microscopically (Leica, Germany) at a magnification of ×100. To quantitatively analyze the area of intact adipocytes and the number of mature capillaries, images in 8 random fields per section from each group (×100 magnification) were examined by using ImageJ software. Immunochemical staining for C/EBP-α (Bioss, China, cat.bs1630R), PPARγ (Bioss, China, cat.bs4888R), Adiponectin (Bioss, China, cat.bs0471R), and CD31 (Sino Biological, China, cat.50408-T16) was performed to determine the extent of adipose tissue and blood vessel formation in new growth tissues at the 4th week.

### Statistical analysis

Each experiment was repeated at least three times. Data were expressed as mean ± standard (SD). Statistical analysis was performed with a paired Student’s *t*-test. A probability (*p*) value < 0.05 was considered statistically significant.

## Results

### Characterization of ADSCs, sEV-LT, and sEV-AT

**Fig. 1:**
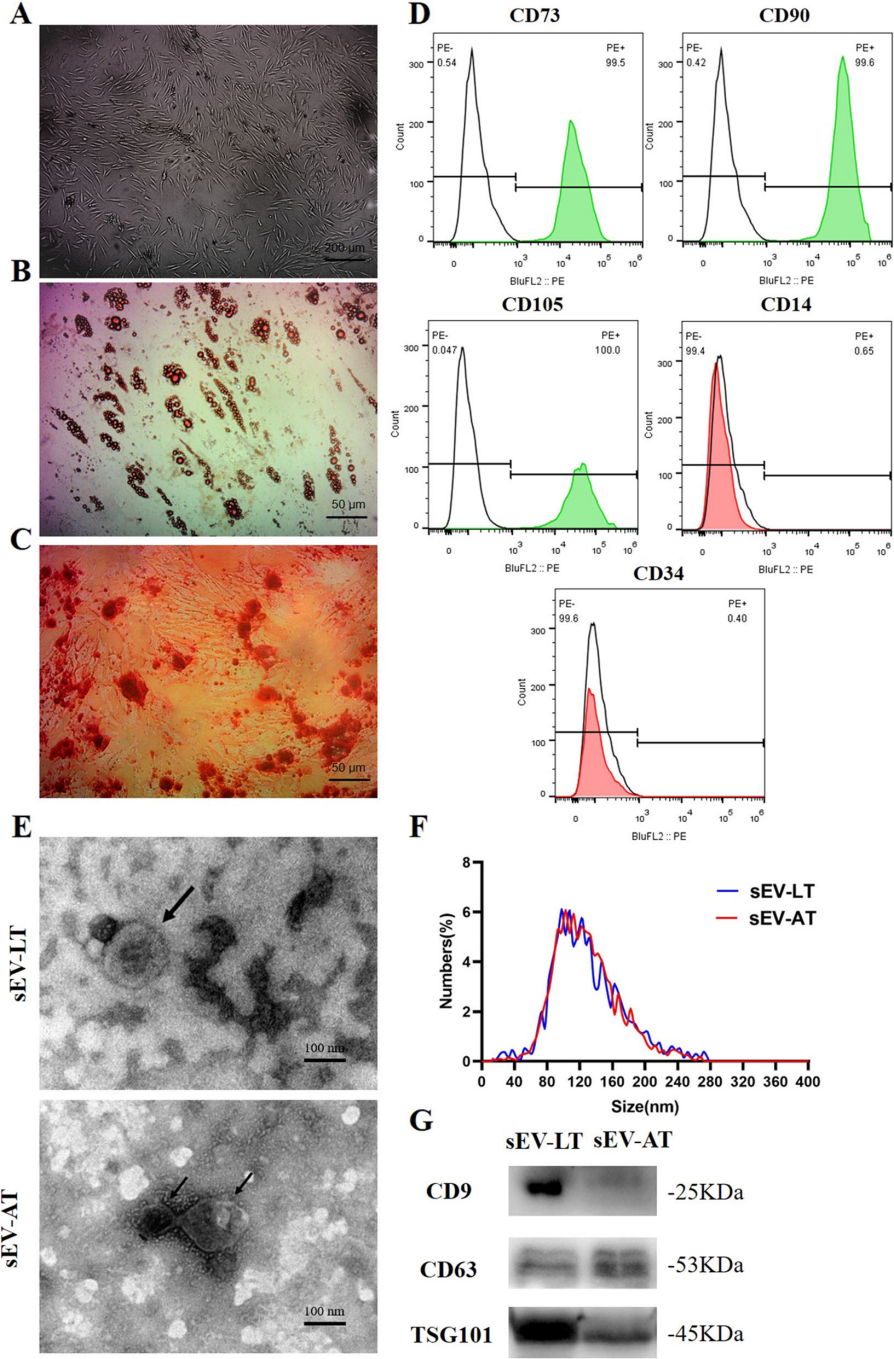
Characterization of ADSCs, sEV-LT and sEV-AT. **a** ADSCs exhibited the typical spindle-shaped morphology, as shown in the picture. Scale bar = 200 μm. **b** Representative photographs of Oil red O staining for adipocytes. Scale bar = 50 μm. **c** Representative photographs of Alizarin Red S staining for osteoblasts. Scale bar = 50 μm. **d** Flow cytometry analysis showed that these cells were highly positive for CD105, CD90, and CD73 but negative for CD34 and CD31. Abbreviations: PE, phycoerythrin. **e** The ultrastructure of sEV-LT and sEV-AT under transmission electron microscopy. Scale bar = 100 nm. **f** The size distribution profile of sEV-LT and sEV-AT by dynamic light scattering. **g** The expression of the sEV markers CD9, CD63, and TSG101 was confirmed by immunoblotting

The morphology of ADSCs at passage 3 exhibited a spindle-like shape which is typical for mesenchymal stem cells under the inverted microscope (Fig. 1a). ADSCs also displayed adipogenic differentiation and osteogenic differentiation abilities, which were demonstrated by Oil red O staining for adipocytes (Fig. 1b) and Alizarin Red S staining for osteoblasts (Fig. 1c), respectively. Flow cytometry analysis (Fig. 1d) showed that these cells were highly positive for CD105, CD90, and CD73, while negative for CD34 and CD31. These results were consistent with previous studies in the characterizations of ADSCs [7]. Therefore, all these data unequivocally confirmed that ADSCs were successfully isolated from human normal adipose tissues.

We then isolated sEV-LT and sEV-AT from the tissue extracts. The cup-shaped morphology of the extracellular vesicles was observed by TEM (Fig. 1e). The size of sEV-LT and sEV-AT were directly tracked by using a Particle Metrix Analyzer named the ZetaView system. The average size of sEV-LT and sEV-AT were 121.3 nm and 121.1 nm, respectively (Fig. 1f). Western blots also showed that the exosomal markers CD9, CD63, and TSG101 (Fig. 1g) were all expressed in sEV-LT and sEV-AT. These results indicated that sEV-LT and sEV-AT were successfully isolated as they were consistent with the defining characteristics of sEV [14].

### The biological responses of ADSCs to sEV-LT and sEV-AT

**Fig. 2:**
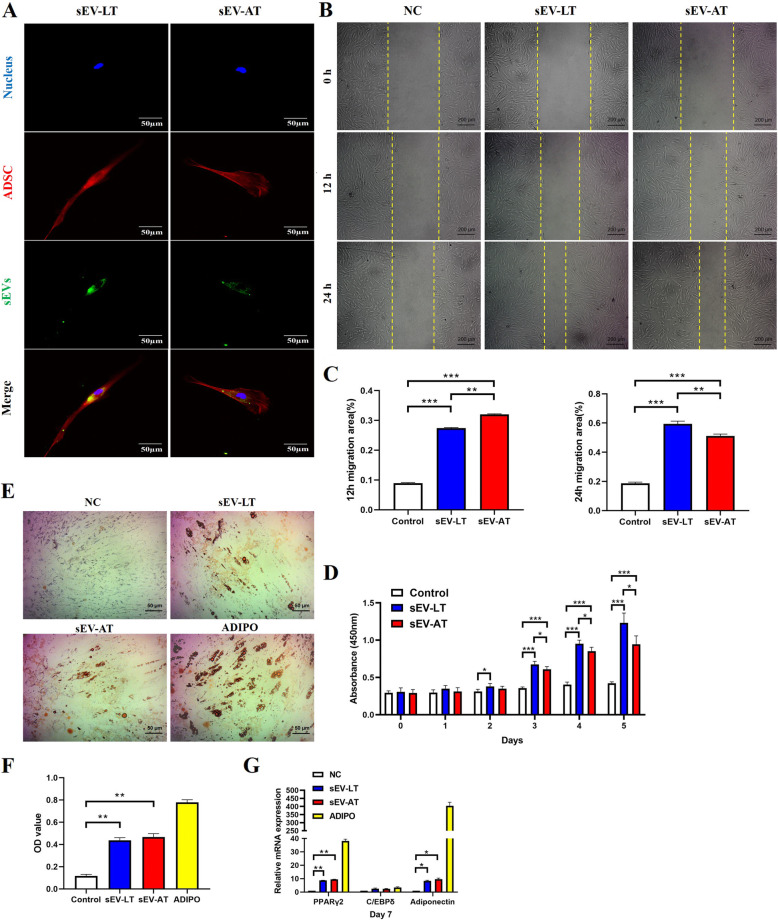
The biological responses of ADSCs to sEV-LT and sEV-AT. **a** The uptake of sEV-LT and sEV-AT by ADSCs. ADSCs were incubated with DiO-labeled extracellular vesicles (green) and stained with phallotoxins (red). Nuclei were stained with DAPI (blue). Scale bar = 50 μm. **b** Representative images from the scratch wound assay. Scale bar = 200 μm. **c** Quantitative analysis of cell migration in each group at 12 h and 24 h (*n* = 3). **d** CCK-8 assay (*n* = 3). **e** ADSCs cultured with sEV-LT and sEV-AT for 14 days, and the lipid droplets were stained with Oil Red O to determine the level of adipogenesis. ADSCs cultured with basal culture medium (negative control, NC) or adipogenic medium (ADIPO) were used as a negative and positive controls, respectively. Scale bar = 50 μm. **f** Quantification of the amount of Oil Red O (*n* = 3). **g** The relative expressions of mRNA encoding C/EBPδ, PPARγ2, Adiponectin was measured by RT-PCR on day 7 after induction. Results are present as mean ± s.d. (*n* = 3). *p < 0.05, **p < 0.01 and ***p < 0.001

To explore whether sEV-LT and sEV-AT could be internalized by ADSCs, ADSCs were co-cultured with the DiO-labeled extracellular vesicles for 6 h and then observed by the fluorescence confocal microscopy. The DiO-labeled (green) sEV-LT and sEV-AT were seen to localize predominantly at the perinuclei region after entering cells, indicating that endocytosis might be the main mechanism through which ADSCs internalized extracellular vesicles (Fig. 2a).

In order to preferably examine the effects of sEV-LT on the migration and proliferation of ADSCs, we used sEV-AT for comparison. It was clear that sEV-LT and sEV-AT promoted the migration of ADSCs at different time points. The results from the scratch closure test demonstrated that the migration of ADSCs increased after 12 h and 24 h in the presence of sEV-LT and sEV-AT compared to the migration in the control group. At 12 h, cell migration of sEV-AT group was higher than that of sEV-LT group. However, cell migration of sEV-LT group was higher than that of sEV-AT group at 24 h (Fig. 2b–c). Likewise, CCK-8 analysis showed that compared to the control group, sEV-LT and sEV-AT at a concentration of 40 μg/mL both increased the proliferation of ADSCs, while sEV-LT had a much stronger promotion effect than sEV-AT (Fig. 2d).

To examine the effects of sEV-LT on adipogenesis, ADSCs were continuously co-cultured with sEV-LT (40 μg/ml) for 14 days. Lipid droplets were showed when ADSCs were treated with both sEV-LT and sEV-AT after 14 days induction (Fig. 2e). Adipogenesis was further determined by Oil Red O staining. The results showed that the OD value of extracted Oil Red O was much higher in both sEV-LT group and sEV-AT group than that in the negative control group. However, there was no significant difference in OD value between sEV-LT group and sEV-AT group (Fig. 2f). Similarly, RT-PCR revealed that the mRNA levels in sEV-LT group and sEV-AT group for the adipogenic genes encoding PPARγ2 and ADIPONECTIN were remarkably elevated on day 7 compared to that in the negative control group, while there was no significant difference in mRNA expression between sEV-LT group and sEV-AT group (Fig. 2g). Moreover, the expression of mRNA encoding C/EBPδ remained at a similarly low level (Fig. 2g).

### sEV-LT promoted adipose tissue regeneration in vivo

#### Gross observations and weights

**Fig. 3:**
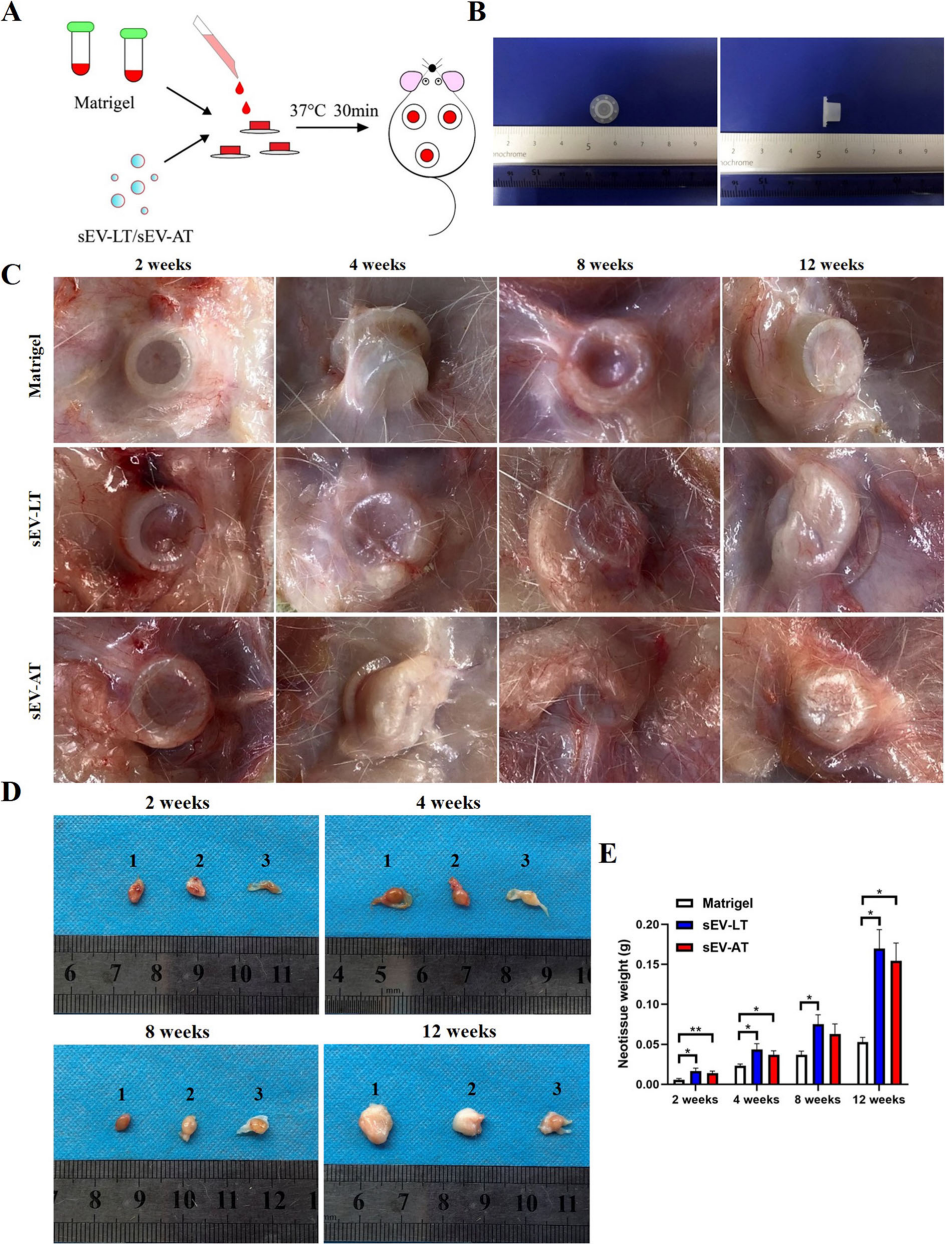
sEV-LT promoted neotissue formation in vivo. **a** The schematic view of the experimental operation process. **b** The implanted silicone tubes, with an internal diameter of 5.0 mm and a height of 5.0 mm. **c** The gross observation of the implanted Matrigel mixed with sEV-LT and sEV-AT within the tube at weeks 2, 4, 8, and 12 after transplantation. **d** Dimensions of neotissues at weeks 2, 4, 8, and 12 after transplantation. (1) sEV-LT. (2) sEV-AT. (3) Matrigel group. **e** Weight comparisons of neotissues at weeks 2, 4, 8, and 12 after transplantation (*n* = 4). *p < 0.05, **p < 0.01

In order to explore the effects of sEV-LT on adipose tissue regeneration in vivo, Matrigel mixed with sEV-LT and sEV-AT was injected into the silicone tube and kept at 37 °C for 30 min to become solid, then transplanted into the back of SD rats, respectively, while the Matrigel alone served as the control (Fig. 3a). The gross appearance of tubes was shown and their appearance at 2, 4, 8, and 12 weeks revealed active angiogenesis and adipogenesis in the sEV-LT group and sEV-AT groups, since there were a few growths of capillaries and adipose-like tissue from host into the tubes, while in control group, tubes filled with Matrigel were mainly encapsulated by integral fibrous fascia (Fig. 3b, c). The neotissues in the tubes were harvested and the macroscopic images at all time points were taken (Fig. 3d). The weights of neotissues were systematically calculated at each time-point and found gradually increased in all groups. Furthermore, the weights of neotissues were significantly increased in the sEV-LT group and sEV-AT group compared to the control groups, but there was no significant difference in that between sEV-LT group and sEV-AT group (Fig. 3e).

#### Histological observations and newly formed capillaries

**Fig. 4:**
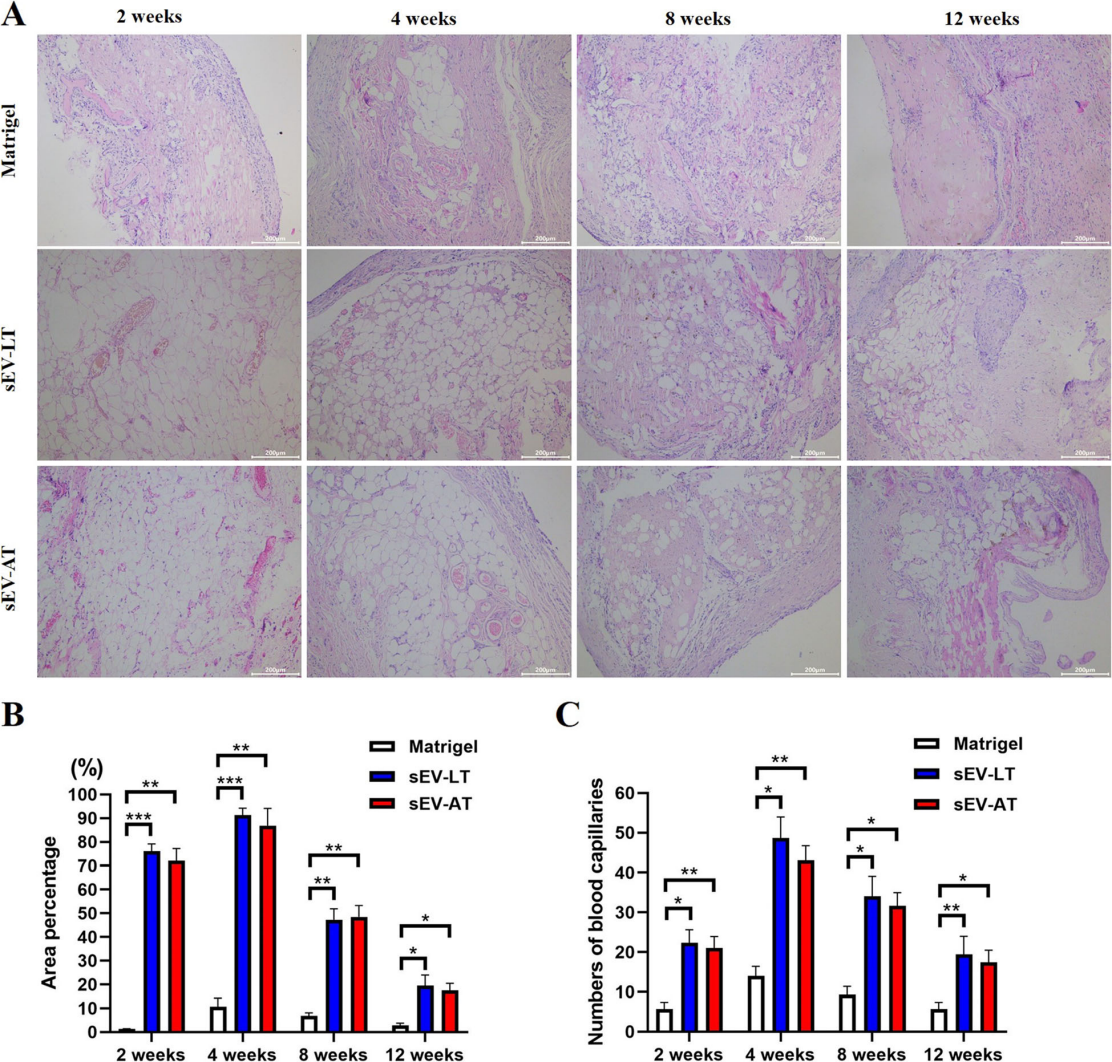
sEV-LT promotes adipose tissue regeneration in vivo. **a** Histology of Matrigel group, sEV-LT group, and sEV-AT group sections at weeks 2, 4, 8, and 12 after transplantation. Sections were stained with hematoxylin and eosin. **b** Comparison of the average adipocyte areas in experimental and control groups at 2, 4, 8, and 12 weeks after transplantation (*n* = 4). **c** Comparison of the mean capillary numbers of neotissue sections in the experimental and control groups at 2, 4, 8, and 12 weeks (*n* = 4). Scale bar =100 μm. *p < 0.05, **p < 0.01, ***p < 0.001

Neotissues from all groups were stained with hematoxylin and eosin (H&E), and the quantitative comparisons of the newly formed areas of adipocytes and capillary numbers have been carried out. In the sEV-LT group and sEV-AT group, light microscopy revealed that immature adipocytes with irregular morphologies and small diameters showed up after 2 weeks’ transplantation, and neovascularization was seen beside the adipocytes, while no obvious adipocytes grew in the Matrigel group (Fig. 4a). At the 4th week, the light microscopy showed that most of the Matrigel contained in each group had been absorbed completely, and an increased large number of regular adipocytes and capillaries were seen in the sEV-LT group and sEV-AT group (Fig. 4a–c). At 8 and 12 weeks, the adipocyte areas and the numbers of capillaries observed in the sEV-LT group and sEV-AT group decreased, while the granulation and fibrous tissue areas gradually increased (Fig. 4a–c). Although some of the adipocytes were replaced by fibroblasts and granulosa cells, the remaining were maintained for 12 weeks and there still had significant increase in adipose tissue in the sEV-LT group and sEV-AT group than the Matrigel groups (Fig. 4b, c).

#### Immunohistochemistry

**Fig. 5:**
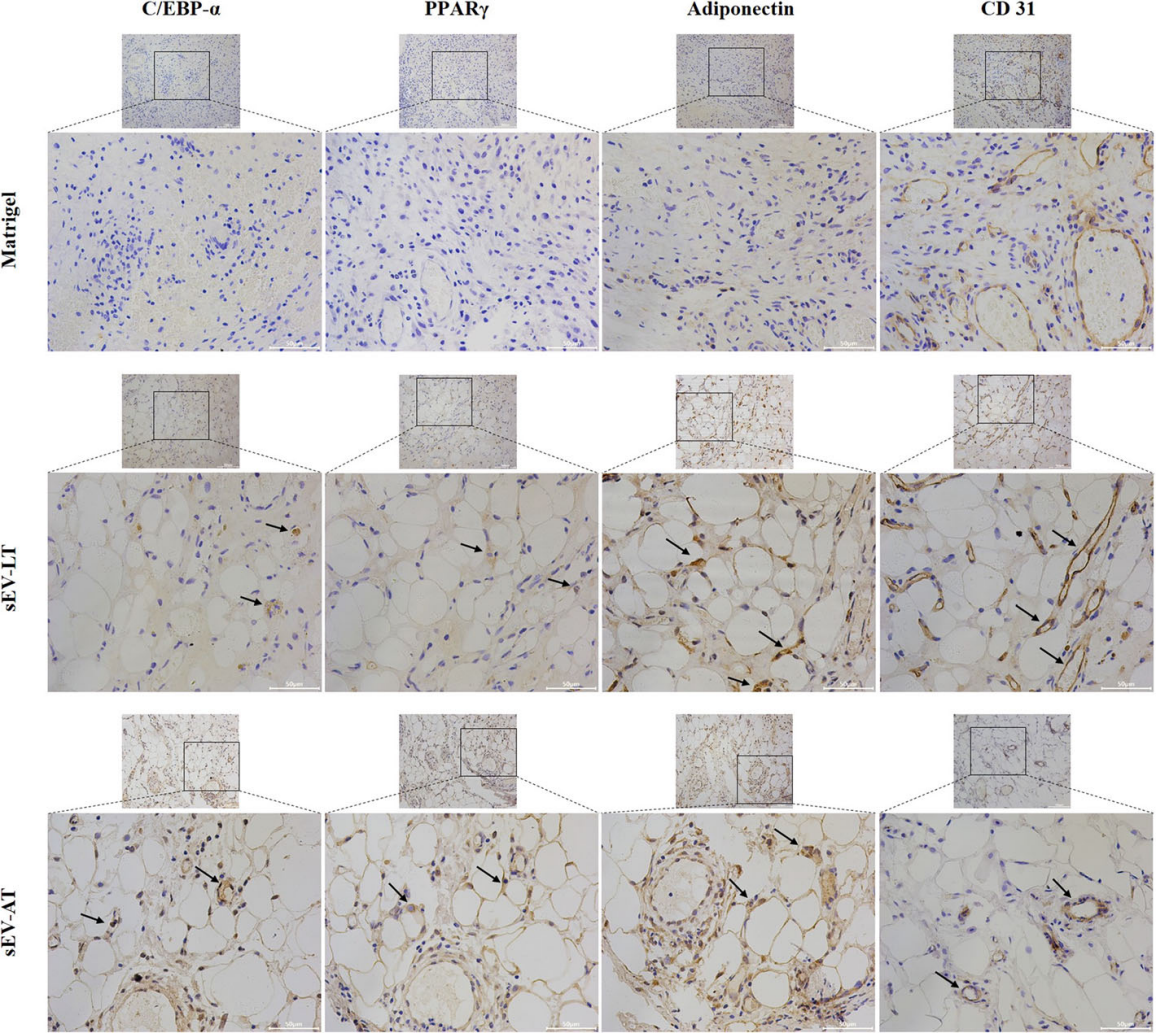
Immunohistochemistry of neotissue sections in the experimental and control groups at week 4 after transplantation. Representative photographs of C/EBPα, PPARγ, Adiponectin, and CD31 immunostaining in the Matrigel group, sEV-LT group, and sEV-AT group, respectively. The black arrows indicated the positive stained protein. Scale bar = 50 μm

Furthermore, we used immunohistochemistry to ascertain the adipogenesis and angiogenesis effects of sEV-LT. Neotissue sections at the 4th week were stained with antibodies against C/EBP-α, PPARγ, Adiponectin, and CD31, which were all positively expressed in the cells of sEV-LT group and sEV-AT group. However, in the Matrigel group, only positive expression of CD31 protein was found. In addition, we found that many adipocytes and endothelial cells were positively stained near the blood vessels, while those cells located at a distance from the blood vessels were negative (Fig. 5).

## Discussion

Previous studies in cell-free therapeutic approaches for adipose tissue regeneration have demonstrated certain effects to contribute to new adipose tissue development, including using synthetic or natural biomaterial scaffolds loaded with adipogenic growth factors, or providing a microenvironment suitable for originally existing cells in the body to migrate, proliferate, and differentiate to form adipose tissues [22, 23]. However, problems such as immune rejection responses or potential side effects of high growth factor concentrations may make it difficult to apply in clinic extensively. In recent studies, researchers have found and confirmed that cell-free adipose tissue extract (ATE) could effectively induce adipogenesis and angiogenesis [5, 24]. Furthermore, sEV-AT, as an indispensable component of ATE, has also been found that it could modulate proliferation, migration, and differentiation of the target cells by transferring functional proteins, mRNAs, and miRNAs [16, 25]. For example, sEV-AT under hypoxic conditions might promote lipid stimulation in 3T3-L1 adipocytes by increasing the levels of lipogenic enzymes, including fatty acid synthase (FASN), glucose-6-phosphate dehydrogenase (G6PD), and acetyl-CoA carboxylase (ACC), which might contribute to adipose tissue homeostasis or dysfunction [26]. In addition, Zhang et al. [17] found that sEV-AT could induce adipogenesis differentiation through a mechanism involving transfer of miR-450a-5p. Meanwhile, miR-450a-5p could promote adipogenesis through repressing expression of WISP2 by targeting its 3′ untranslated region. To conclude, from a perspective of paracrine effect, sEV-AT could be delivered into target cells and could give rise to adipogenesis ultimately. Lipoma tissues, as adipose tissues of benign tumors, were always defined as useless tissues in the application of regenerative medicine and tissue engineering [27]. Previous studies basically focused on lipoma-derived stem cells; however, the exact biological functions of sEV-LT are still needed to be found and determined [28, 29].

Our study was among the first giving details in the characteristics and adipogenic analysis of sEV-LT and comparison with sEV-AT from human. In our studies, we showed evidence that lipoma tissue could release sEV and ADSCs could uptake both sEV-LT and sEV-AT in a form of endocytosis. Furthermore, we found that sEV-LT and sEV-AT were able to effectively promote the proliferation, migration, and adipogenic differentiation of ADSCs. Compared with sEV-AT, sEV-LT had a stronger ability to promote the proliferation and migration of ADSCs, while there was no significant difference in inducing adipogenic differentiation of ADSCs. These results indicated that the local progressive growth of the lipoma tissue might be related to the increasing migration and proliferation of the surrounding ADSCs induced by sEV-LT. In fact, previous studies have also confirmed that EVs secreted by terminally differentiated cells can influence biological functions of stem cells [30–32]. For instance, EVs from osteoblasts, vascular endothelial cells, and skeletal muscle cells were found to be involved in the regulation of osteogenic differentiation, angiogenic differentiation, and myogenic differentiation of stem cells, respectively [30–32]. Once the stem cells differentiate into mature cells, they, in turn, regulate stem cells to differentiate through EVs; thus, a positive-feedback loop mechanism is formed during differentiation. Therefore, both sEV-LT and sEV-AT presented similar effects on ADSCs. However, we observed that sEV-LT showed stronger effects in promoting proliferation and migration of ADSCs than sEV-AT; these findings should be related to the differences in the functional proteins and miRNA and other genetic materials contained in the two kinds of sEV, which may be associated with the core reason for the tumorigenicity of lipoma tissue.

In order to further investigate the potential functions of sEV-LT in adipose tissue regeneration, we transplanted sEV-LT into subcutaneous chamber models of SD rats and observed the effects over a long-term period of 12 weeks, which was sufficient for complete adipogenesis and neotissue stabilization. We found that capillaries were first visible at 2 weeks on the surface of the silicone tubes in the sEV-LT group and sEV-AT groups, while none of that were seen on the surface of the cannula in the Matrigel group. At 2, 4, 8, and 12 weeks, the adipocytes were detected under the microscope in the sEV-LT and sEV-AT groups and mainly appeared around the newly formed blood vessels. Compared with the Matrigel group, the adipocyte areas and the number of capillaries in the sEV-LT and sEV-AT groups were increased and statistically significant at all time points. Collectively, these results showed that the sEV-LT were able to promote adipogenesis and angiogenesis in vivo and the positive regulation relationship between adipogenic differentiation and angiogenesis, which was consistent with previous studies. It has been shown that the increased resorption of a fat graft is often related to debilitated and weakening neovascularization [33]. Vascular-derived factors regulate the metabolism and accumulation of adipose tissue by affecting the formation and reconstruction of adipose tissue vascular network [34]. Due to the close relationship between adipogenesis and angiogenesis, we used immunohistochemistry to analyze the expression of adipogenesis-related proteins PPARγ, C/EBP-α, and Adiponectin and angiogenesis-related protein CD31 in each group at 4 weeks. It was found that all these proteins were positively expressed in the sEV-LT and sEV-AT groups, and adipogenesis-related proteins were mostly aggregated and expressed in adipocytes around capillaries. In contrast, they were relatively less expressed in adipocytes far away from microvessels. These results also confirmed the positive regulatory relationship between adipogenesis and angiogenesis.

However, along with the decrease of blood vessels, the adipocytes recruited in the sEV-LT and sEV-AT groups began to decrease and were replaced by fibroblasts and granular cells at 8 and 12 weeks. We hypothesized the main reason could be related to the rats’ immune responses to those silicone tubes and limited quantities of EVs which gradually exhausted. Nevertheless, from a long-term perspective, the newly formed adipose tissue in sEV-LT could still display a certain vitality till 12 weeks, which indicated a therapeutic potential of sEV-LT in fat regeneration and tissue engineering.

There are some limitations of our studies, and several problems must be solved before the results can be applied in the clinic setting. For instance, although we have verified the adipogenesis and angiogenesis effects of sEV-LT, the exact proteins or miRNAs involved in sEV-LT-mediated differentiation are still needed to be determined in the future. Another limitation is that we need to further evaluate and confirm the tumorigenicity and biosafety of sEV-LT, since they are extracted from tumor tissue. Moreover, the number of SD rats in the study was too small; a larger sample size should be included in the future study. Additional studies will be performed in the future; however, our studies have provided a potential new method for adipose tissues regeneration and these findings will direct better clinical treatment.

## Conclusion

In this study, to the best of our knowledge, we provided direct evidence that sEV-LT exerted promotion effects on adipose tissue regeneration by accelerating the proliferation and migration and adipogenic differentiation of ADSCs in vitro, recruiting adipocytes and promoting angiogenesis in vivo for the first time. Notably, the neotissue induced by sEV-LT could maintain viability lasting for 12 weeks. Therefore, sEV-LT could serve as an alternative cell-free therapeutic strategy for generating adipose tissue, providing a promising application prospect in tissue engineering.

## Data Availability

The data that support the findings of this study are available from the corresponding author upon reasonable request.

## Abbreviations

ADSCs: Adipose tissue-derived stem cells
ACC: Acetyl-CoA carboxylase
ATE: Adipose tissue extract
H&E: Hematoxylin and eosin
FASN: Fatty acid synthase
G6PD: Glucose-6-phosphate dehydrogenase
LTE: Lipoma tissue extract
MSCs: Mesenchymal stem cells
PBS: Phosphate-buffered saline
sEVs: Small extracellular vesicles
sEV-AT: sEVs derived from the adipose tissues
sEV-LT: sEVs derived from the lipoma tissues
TEM: Transmission electron microscopy
